# Navigating Detransition Borders: An Exploration of Social Media Narratives

**DOI:** 10.1007/s10508-023-02556-z

**Published:** 2023-02-23

**Authors:** Tait Sanders, Carol du Plessis, Amy B. Mullens, Annette Brömdal

**Affiliations:** 1 School of Psychology and Wellbeing, Faculty of Health, Sciences and Engineering, Centre for Health Research, Institute for Resilient Regions, University of Southern Queensland, Ipswich, QLD 4305 Australia; 2 School of Education, Faculty of Business, Education, Law and Arts, Centre for Health Research, Institute for Resilient Regions, University of Southern Queensland, Toowoomba, QLD 4350 Australia

**Keywords:** Detransition, Trans/transgender, Livable lives, Trans architecture, Social media

## Abstract

Detransition, a relatively recent phenomenon within academic discourse and mainstream media, refers to individuals who transition from the gender they transitioned into. Experiences of detransition, including those shared on social media, are poorly understood. Drawing upon narratives of gender detransition as shared on a global social media site, this analysis explores and seeks to better understand how detransition experiences are shared; and the effect of detransition narratives on gendered embodiment and belonging. Employing Butler’s (Undoing gender. Routledge, 2004) notion of livable lives and Crawford’s (Seattle J Soc Justice 8(2):515–539, 2010) conception of trans architecture, this analysis theoretically extends trans conversations to include discourses and narratives of detransition. A total of 130 archival posts by 36 contributors relating to detransition were collected from a popular global social media site where the engagement of reflexive thematic analysis contributed to the development of three themes: Contemplating transformation; Experiences of detransition; and Prominent discourses for detransition. Study findings suggest that detransition narratives expressed on this social media site demonstrate the multifaceted and complex ways in which non-normative gendered lives are rendered unlivable. In response, this analysis problematizes gender by conceptualizing detransition as a transformation toward a trans space outside a cisnormative frame contributing to making gendered lives more livable.

## Introduction

Transitioning and detransitioning gender share some similarities as both begin with a movement from a normative to a non-normative gender and body. This initial transition process may involve corporeal and/or social changes such as those produced through gender-affirming surgeries and therapies, including changes to legal name and preferred pronouns (Shepherd & Hanckel, 2021). It is at this supposed resting place of gendered stasis, post-transition, that those who undertake actions to be recognized as a gender other than that represented by the transitioned gender and body, appear to depart toward more normative gendered formations.

Nomenclature regarding detransition is currently in flux and contested, with a majority of scholars positing detransition as a reverse or return to the gender presumed at birth (see Dhejne et al., 2014; James et al., 2016; Marchiano, 2017, 2021; Turban et al., 2021; Watt, 2019, 2020; Yoo, 2018). A number of recent studies advance alternate definitions of detransition to include cessation of transition and/or trans identity by way of intentional changes to gender-affirming therapies and/or surgeries and/or social attributes that do not specifically require a return or reversal to the gender presumed at birth. For example, Vandenbussche (2021) uses the terms “(male or female) ‘detransitioner’” (p. 1603) to describe participants who may have ceased or altered medical and/or social attributes, and who intentionally identify themselves “as a detransitioner” (p. 1603) but does not include the requirement of a person to reverse or return to the gender presumed at birth post-detransition. Here, the terms male and female related to detransition were used to reflect how participants self-identified within the survey by Vandenbussche (2021). In Littman’s (2021) survey of 100 participants, it reflects that “detransition is the act of stopping or reversing a gender transition” (p. 3353) and uses the terms “natal males” and “natal females” to describe the recruited individuals, and the survey also does not require detransition to incorporate a return to the gender presumed for them at birth. Littman additionally comments on the contested nature of terminology and the use of natal sex when referring to the participants. Likewise, Guerra et al. (2020) in their case study of four participants, define detransition synonymously with retransition as “reversal of changes made to the gender reassignment process, whether medical, social or administrative… [that] may or may not be associated with identity desistance” (p. 564). By identity desistance, Guerra et al. (2020) refer to a change in “sense of identity” (p. 565) or a return to the gender presumed at birth. Last, Expósito-Campos (2021) formulates a typology of detransition to include two types revolving around the cessation or continuance of a trans identity. The first type, called “core gender detransition” (p. 272), involves ceasing a trans identity and “identifying with his/her birth sex” (p. 272). Within this type, trans is situated as an identity that was adopted or applied, sometimes mistakenly or unsuitably, in response to underlying psychological issues or unresolved childhood trauma; or that becomes incongruent with new attitudes or beliefs among other factors. The second type, called “non-core gender detransition” (p. 272), involves the continuance of a trans identity but cessation or reverse of “their gender transition” (p. 272) due to health, medical, social, support, discrimination, and/or economic related concerns among other factors. While these definitions of detransition, based on the lived experiences of individuals within the respective studies, acknowledge that detransition does not necessitate a return to the gender presumed at birth; they are arguably centralized around fixed and stable trans, and/or cisgender identities. As such, they do not engage with the question of how knowledge of detransition relates to dominant cisnormative discourse, and in turn affect how gender is embodied and belonging experienced by those who detransition.

With regard to positionality, the authors of this study include people with lived experience of detransition, and are trans critical thinkers, trans rights and health scholars, trans and detrans allies, and clinicians. As such, the aforementioned factors including our academic positions, and the adversity experienced as members of trans and detrans communities, collectively contribute to our epistemic framework, definitions, analysis, and the subsequent research outputs. For the purpose of this study, the term detransition refers to individuals who have undertaken actions recognized as transitioning gender (e.g., gender-affirming hormone therapy and/or surgeries, changes to name and/or pronoun), and subsequently changed one or more of these social and/or corporeal attributes to be recognized as a different gender. This definition does not envisage a final or fixed intelligible gender identity or gender as a temporal destination or attribute of a person, but rather suggests that we unpack the relations between body, processes, gender, and recognition. Consequently, our definition focused on the actions and associations related to detransition, incorporates detransition to any gender, including the gender designated at birth, as well as no gender.

As such, the definition used in this analysis accords with the work of Pehl (2020) who leveraging Butler (2004) forwards the term detransition “negates [the] gender fluidity of trans people by relying on essentalistic understandings of a ‘true’ gender core” (p. 1). Building on Pehl’s analysis, we also lean on Butler (2004) to call into question the role of dominant normalizing forces (discourses—processes and technologies) such as cisnormativity, e.g., the assumption that a person’s gender matches/correlates with the gender that was presumed for them at birth (Worthen, 2016) in relation to how gender through detransition is recognized, made intelligible, and embodied. We suggest that gender outside recognizable forms such as cisgender are consequently unattainable, undesirable, unknown, and even unlivable (Butler, 2004).

Further to the discourse regarding terminology, in this critical analysis the term trans is also used as per Radi’s (2019) use of the term trans*, to encapsulate the multiplicity of non-cisnormative genders not limited to “trans* women and men” (p. 45). This study, recognizes that cisnormative discourses may result in the destruction of livable lives (Butler, 2004), therefore, the authors make use of the pronouns *they* and *them* when referring to those who share/contribute to the social media site/s explored here, rather than pronouns such as *he* and *she* that are commonly considered to exist only within cisnormative discourse. Individual contributors cited within this paper may however use other pronouns than those we have provided in this analysis, and outside the purpose of this study, we stress the importance of using an individual’s preferred pronouns.

Another point of departure for those who detransition is in the area of access and belonging (or lack thereof) to spaces and sites of sociocultural significance that provide shelter, agency, and connection. Participation in and belonging to gender post-detransition, is arguably problematized by a history with and experiences of dipping into and out of gender normativity. While further research is needed to understand the links between suicide attempts made by trans individuals, currently nine times the rate of cisnormative counterparts in the US (James et al., 2016), and the role of support networks such as broader LGBTIQ communities and allied spaces, arguably support and connection to community may be a protective factor regarding suicidality. Conversely, safe and affirming spaces for those who detransition are comparatively sparse, and social media sites appear integral for connection, information, story, support, and survival. In response a growing number of individuals are sharing their experiences of detransition on social media sites by contributing information, resources and support. These spaces are currently the sites outside academia in which detransition discourses, knowledge, belonging, and body are being constructed.

Of particular interest to our study were the ways in which knowledge of detransition are constructed through social media posts. Within this context, two recent studies explored aspects of detransition and the role social media use played regarding detransition. Vandenbussche (2021) conducted a cross-sectional online survey of 237 participants to explore the needs of “detransitioners from online detrans communities” (p. 1603), and found that engaging with others online about experiences of detransition, sharing their own stories and hearing others, is a crucial form of support in relation to detransition. The second study by Littman (2021) recruited 101 participants to conduct an online survey to provide a description of a population that had detransitioned by “discontinuing medications, having surgery to reverse the effects of transition, or both” (p. 3354). While not explicitly focused on experiences of social media use, this study describes how social media was reported to be influential in encouraging trans identification and transition. Unlike the study by Vandenbussche (2021), Littman’s (2021) study does not explore the role social media sites play in terms of providing support regarding detransition.

Likewise, in Yoo’s (2018) study of 11 individuals, some recruited from a social media site and interviewed about experiences of and with detransition, participants reported being misdiagnosed with gender dysphoria, and that transition was encouraged by and through a “trans narrative” (p. 184). Yoo describes that the term “trans narrative” has two meanings, and the one relevant to this study defines it as a “dominant discourse among sects of the trans/gender-diverse communities that do not allow dissent, disagreement, or alternative views” (p. 184). Within our study, we use the terms *trans ideology* and *trans-affirming narratives* aligning with Yoo’s definition of trans narrative but also extend it to include medical/mental health professionals, families, partners, parents, and friends of trans persons. Diverging from Yoo’s assertion that these groups “do not allow dissent, disagreement, or alternative views” (p. 184), our study uses the terms to describe discourses that champion gender-affirming transition and processes.

The idea that trans-affirming narratives are enforced is also commonly heard from trans-exclusionary radical feminists (TERF) and Christian lobby groups within mainstream media and academic settings alike, further highlighting the conservative and contentious social and political climate surrounding trans and detransition (Pearce et al., 2020; Slothouber, 2020; Tudor, 2021; Williams, 2020). Guerra et al. (2020) in their recent case study of four participants who detransitioned raise a notable point regarding trans-affirming narratives in relation to the role of media. First, they suggest that “excessive and inadequate media exposure” targets “all people with gender variants towards medicalization” and “might generate strongly binary models” (p. 566). Further, Guerra et al. (2020) argue that “guidance limited to the testimonies of other transgender individuals in the social media, without other options being explored, can be counterproductive” (p. 566). These arguments suggest that people who access media regarding trans and transition are (1) met with trans-affirmative messages from trans individuals that promote gender-affirming surgeries and/or hormones; (2) are susceptible to being influenced to transition; and (3) that transition via “medicalization” may lead to the erasure of gender variance in favor of binary gendered outcomes. Within this logic, transition via medical means ties the body, actions, and gender to cisnormative discourse. Following this line of reasoning, similarly, social contagion is flagged in research particularly regarding youth who transition gender. Littman (2018) proposes that, like anorexia, gender dysphoria as an affect or behavior, may be contagious for “vulnerable individuals” (p. 5). Although discourses within the space of youth is outside the scope of our study, Watt (2019) also proposes youth are vulnerable to social contagion and Marchiano (2017) has explicitly stated “teens were coming out as trans in peer clusters” (p. 346). While the research showing how behaviors may be influenced due to online social media use and social or peer contagion is broadly valuable, we contend that it is problematic in relation to trans and detransition, as it assumes a vulnerable and cisgender population. In this discourse, individuals are positioned as having a “fragile” (Watt, 2019, p. 16) sense of cisgendered identity with an “innate vulnerability” (p. 16) of being influenced to take on a trans identity. Conversely, we would suggest that when people access social media, they are supported by others to embrace their transness, which may result in the person undertaking gender-affirmative processes and care.

To promote an alternate understanding in support of gender transformations, this study extends Butler’s (2004) notion of livable life into the space of detransition, positing that some who transition gender experience gender to be unbearable post-transition in the face of unattainable gender norms. To gain a more livable life, a number of individuals who decide to detransition gender are in a sense un- or re-doing one or more of the attributes changed during their gender-affirming transition (social, legal, and/or physical). Closely related to Butler’s theory of livable life, this paper also extends Crawford’s (2010) concept of trans architecture by imagining detransition to encompass a trans space outside a cisnormative frame. Here, alternate ways of constructing gendered homes contribute to making gendered lives livable.

To this end, this analysis aims to explore how detransition narratives shared in online social media spaces contribute to knowledge about and lived experiences of detransition. Dedicated to capturing the voices and narratives of detransition, this critical analysis is framed by two research questions exploring: (1) what are some of the narratives about detransition shared in social media posts; and (2) in what ways may detransition narratives challenge and/or affirm normative gender?

Within a social and political landscape of cisnormative narratives, the authors seek to broaden understandings to affirm the ways in which those that contemplate and/or undergo gender transformations can meaningfully enhance their livable lives (Butler, 2004). The outcome of this analysis will likely be of significant interest to those who navigate gender borders, including health practitioners in the support of those who traverse these boundaries.

## Method

### Theoretical Framework

Gender transition is often posited as being motivated by a desire to locate a gendered home that fits more comfortably than the gendered temporal location (e.g., where the body is located in relation to a particular discourse at a particular point in time) currently inhabited (Pehl, 2020; Prosser, 1998). This absence of a comfortable home may be experienced as feeling unsafe, lack of belonging, and even feelings of distress about the shape and form of the body, and its failure to represent a person’s gender. Transition in this way may be viewed as seeking a more suitable and fitting gendered space and belonging. For those that transition and then contemplate or proceed with detransition, it is possible that the expected sense of gendered home was not found. Put differently, Butler (2004) proposed the concept of a livable life as being a life made possible (reified), and given agency through a process of being recognized. In simplified terms, recognition is achieved when a life resembles that which we know to be recognizable. As dominant gender discourse is based on cisnormative conceptions, our understanding of how gender is valuable and productive is formed through this lens. Where gender falls outside this knowing, it ceases to exist, and becomes unintelligible and unrecognizable. In cisnormative discourse trans identities are also often made recognizable through normative conceptualizations and regulation (e.g., through binary genders such as male and female; binary gendered physical spaces such as bathrooms, education settings, and prisons; and binary gendered corporeal and cultural spaces such as bodies, clothing, pronouns, and expected behaviors). Even trans identities such as agender or gender nonconforming, it could be argued, are made visible/given life when interpreted through the vision of a recognizable cisnormative frame. Butler (2004) has further asserted where the conditions/categories of recognition constrain ways of being, “unbearable life or, indeed, social or literal death” (p. 8) are produced and present.

In this analysis, Butler’s (2004) concept of a livable life is extended regarding gender detransition. While not all detransitioners relate to the terms *trans* or *gender* in the same way, we suggest that for some, gender post-transition is made unintelligible and therefore problematic and unlivable. How this un-livability is experienced may be varied but may include a greater risk of violence, socioeconomic deprivation, and reduced access to education, employment, supports, and connection (James et al., 2016; Turban et al., 2021). Furthermore, the collective effect of lived experiences of unrecognizability may result in feeling isolated or a sense of not belonging or feeling at home both within one’s skin, and broader gendered society (Littman, 2021; Turban et al., 2021). Instead of risking inevitability, imagining conditions that promote livable flourishing gendered lives is imperative through a call to action—socially and politically. To this end, Butler (2004) has proposed that “the critique of gender norms must be… guided by the question of what maximizes the possibilities for a livable life” (p. 8).

For Butler (2004) gender is un/de-gendered, metaphorically rendering gender homeless, where Crawford (2010) has imagined an architectural vision for articulating trans and in doing so maximizes the possibilities for a flexible, flourishing home outside the cisnormative system/frame. Drawing on Prosser’s (1998) concept of home, Crawford (2010) has posed that the trans body is tied to ideas of ownership, stasis, permanency, and a “capitalist, middle-class, and heteronormative” (p. 526) space. Crawford (2010) further suggested “try as we might, we will never get home—no surgery or bodily modification will return us to our ‘commencement,’ or to a final resting place of selfhood” (p. 534). In reframing the concept of *being at home*, Crawford (2010) instead proposed the trans body be envisioned as a disruptive and transformative discursive space, a structure for deregulated movement resulting in continuously unique and shifting gendered boundaries. Crawford does not suggest gender becomes homeless, that surgeries/hormones have no place, or that there are particular and correct genders or pronouns, but has proposed our recognition of gender incorporate and acknowledge these complexities. Using this approach, exploring experiences of detransition means envisaging gender beyond movement or reification brought about in opposition to or as a part of a cisnormative system. While the goal and outcome of detransitioning may often appear toward a cis gender, the approach used in this study posits that transitioning and detransitioning gender are not one-way voyages toward a fixed, intelligible, and recognizable gender destination.

### Data Source

As such, this study adopts a qualitative approach, specifically centered around a constructionist paradigm to explore detransition narratives of contributors to a global social media site. Within this paradigm, reality is understood to be subjective, relative, and constructed through social contexts and mechanisms (Neimeyer & Levitt, 2001). The objectives of this paradigm seek to interpret the social rules and norms that underlie and construct the meanings and values of a culture, in this case, detransition (Neimeyer & Levitt, 2001). To effectively address the research questions, this critical analysis draws on archival posts in English made by contributors on an international and publicly available social media platform that could be read by anyone, including those without a social media site membership or account. Site contributors could read others’ posts, initiate their own post, or reply to that of someone else, on a unique topic initiated by a contributor. Narratives in the form of posts rather than direct participant engagement make up the data source of our study, and uniquely so on the topic of detransition. The sample includes all text, images, and videos posted to a specific group discussing the topic of detransition over a 16-day-period during 2019. The overall sample included all posts and subsequent comments made in response to initial posts during the time period on the global social media site. Although all comments on the initial posts were collected, only the initial post of each thread was included in the analysis phase due to the large volume of data. This approach focused on the lead narratives in the time period specified while reaching saturation and maintaining dataset manageability considering the overall dataset size, resources and project time constraints.

The subset of data included in this analysis is made up of 130 initial social media posts by 36 contributors, containing a total of 30,111 words with an average post-length of 230 words about stories of detransition. Due to the static and archival nature of the data, determining a contributors’ relationship to detransition was based where possible on self-identification or otherwise assumed based on the content of their post. Contributors became wide and varied from this vantage, and for the purposes of highlighting the myriad of voices within this study a number of identities/classifications were established. As such, those who contributed to the detransition threads may not agree with the classification they have been allotted. The 36 thread contributor identities that have been classified for this study include (1) detransitioners—19 contributors; (2) those contemplating detransition—9 contributors; (3) those considering transitioning—2 contributors; and (4) parents, partners, and allies of the three aforementioned groups—6 contributors.

### Measures

Two key ethical issues regarding the use of the data in this study were privacy and informed consent. Regarding privacy, current debate suggests ethical use of publicly available social media data without informed consent is possible when guided by a set of principles designed to protect the privacy of the authors contributing the data, do no harm, and minimize risk to the data contributors/authors, and broader community base (Sugiura et al., 2017). Following the lead of other recent research using publicly available online data (e.g., Caruso & Roberts, 2018; Snee, 2013), consent was not sought from the authors/contributors of the social media data used in this study for two reasons: (1) some contributors were no longer active on the social media platform and could therefore not be located; and (2) as the data were collected three years prior, we believed seeking consent may possibly cause psychological harm or stress considering that contributors would need to redisclose/relive their detransition experiences. Parallel to this, guidelines outlined by Hewson (2016) were employed to deidentify the social media contributors from the data used in the study, including, not disclosing the social media site; removing identifiable information (e.g., usernames, age, and occupation); and, presenting stories through themes rather than in a case study format. To further protect the social media contributors from inadvertent identification, a combination of paraphrasing and direct quotations was used to support themes, and pseudonyms were developed. All study activities were approved by the research team’s university ethics committee—University of Southern Queensland’s Human Research Ethics Committee (H19REA237).

### Data Analysis

Braun and Clarke’s (2006, 2019) revised six-phase reflexive thematic analysis was then used to make meaning of the data. An iterative inductive and deductive approach was employed, where the content of the data together, with our theoretical frameworks and authors’ interpretations guided, the code and theme development (Braun & Clarke, 2006, 2019). The process involved the lead author first reading the initial post of each thread collected during the time period, noting key ideas and features, collating codes, and generating themes in collaboration with the rest of the research team. Relevant data were then reviewed for suitability and alignment with the themes. Revision of the themes occurred after review to better address and align with the research questions. Subsequently, themes were named providing a description for each of the narratives within the dataset.

As a result of the analysis, three themes were developed: Contemplating transformation; Experiences of detransition; and Prominent discourses for detransition, and eight secondary themes. We now offer our critical analysis of the gender detransitioning themes generated and defined in this paper.

## Results

### Contemplating Transformation

This first theme contains two sub-themes: *Contemplating Detransition,* reflects experiences shared by those contemplating gender detransition; and *Contemplating Transition,* reflects more normative gender transformation experiences shared by those contemplating transition to a gender other than that presumed for them at birth.

#### Contemplating Detransition

Those contemplating detransition predominantly shared challenges of living in their transitioned gender, interest in others’ experiences, and sought support with making decisions and requesting information about detransition. Some shared thoughts of not feeling or believing themselves to be trans. For example, Jamie expressed “I just don't think I am trans anymore” whereas Harper stated “I realized I'm not trans, [and]… some of my fundamental beliefs about identity [have] completely changed”; while Nicky voiced, “I have realized I am not female to male.” The majority of contributors expressed the belief they were not, and could never be, the gender they had transitioned into and instead believed themselves to be the gender presumed for them at birth. Jamie said “I [do not] think I am trans; I am biologically a man and could never be female. I do not want to be a woman anymore”, and echoed by Lennon expressing, “I just want people to see me as a woman again, as a person again, and not trans.” Alternatively, some contributors expressed they were contemplating detransition to a gender other than that presumed for them at birth, usually a variant of non-binary gender: “I am AFAB [assigned female at birth] non-binary and feel like I have both a male and female body” (Sacha). Those contemplating detransition also posted experiences of feeling *other* to the gender presumed for them at birth, as well as the gender transitioned into:

I do not know what to do anymore, I feel like I am losing my mind sometimes… The problem is when I feel dysphoric, I [become] really suicidal about being… presumed female at birth and feel like my body does not belong to me... Is it possible to detrans[ition] while dysphoric, or do I need to accept that [transitioning] is the only treatment? (Jules)

Some contributors, like Jules, appeared extremely distressed by their experiences post-transition, expressing they felt suicidal and sharing seemingly out of desperation, and seeking support. Within this context Kim stated, “I want to just end it all because I am so stuck in this horrible life” while Jessie voiced, “I thought transition was the answer to all my problems… but it made everything worse… I am suicidal many times a day.” Adding to the distress experienced, many contemplating detransition were concerned with corporeal changes post-detransitioning and sought knowledge of what to expect. Questions about ceasing hormones; impacts to bone density; and size/shape/function of genitals and breasts were commonly expressed by contributors: “What happens when you go off hormones? How long does it take for changes to happen?” (Jordan); and Ashley wondered if their body would revert to a “male” body, specifically, their genitalia: “will it go back to its original size? Will I produce sperm again?” Evident within these post-transition stories were examples of experiences of extreme distress, a need to share lived experiences, and access information and support.

#### Contemplating Transition

While the majority of contributors posted about detransition, others posted about desires to transition. Contributors contemplating transition expressed concern about making a mistake by transitioning, the social media site provided a place for deliberation and discussion with those who had experienced detransition, such as Andy. Here Andy expressed they were a questioning trans person who did not want to make a mistake and hoped that educating themselves about detransition would help them understand what was right for them. Andy’s primary concern before transitioning was regretting it; the thought of detransitioning was very terrifying to them. While regret can be conceptualized as feeling as though the wrong decision was made to transition, this is not to assume the alternate to transition is necessarily to remain in stasis within the (unlivable) gender presumed at birth. As emphasized by Shannon, contemplating transition appeared to involve deliberating how to live with the gender presumed for them at birth rather than to transition:

I cannot continue on as a woman… I have recently come to a turning point… I know medical transition is not some magic solution… I realize that by transitioning, I would only become trans and not biologically the gender I was trying to resemble… [I am] so scared… of continuing to live as my birth gender. (Shannon)

Notably distress was associated with living as the gender presumed for them at birth; and feeling unable to continue to live. It was also clear they believed that medical intervention may not produce a fix for the problem. Where many contributors expressed what could be interpreted as feeling highly disturbed by their overall experience of living with the gender presumed at birth, others such as Kelly expressed a distinct distress about their bodily form: “My physical body… bothers me most… I just want to be normal but I keep thinking about how much I hate my body.” Additionally, Kelly sought information about how other detransitioners thought about and related to their bodies post-detransition. Sentiments such as “I just want to be normal” (Kelly) are indicative of common narratives within cisnormative and medical discourse; that trans bodies are abnormal and other underlying psychological or corporeal health issues exist. Psychosis, childhood abuse, and hormone imbalance were commonly referred to within these discourses. The consequence of this line of reasoning suggests that rather than transitioning gender, the answer lies in addressing underlying mental or physical health concerns; resulting in a literal eviction or evacuation of the trans body.

A smaller number of contributors shared their experiences of considering transitioning but choosing not to. Corey shared a meme describing a series of ways to deal with living with the gender presumed for them at birth without transitioning, including identifying how dysphoria manifests and what triggers it, and using strategies such as talking to others and doing art. In a similar vein, Reese shared their gratitude for not starting hormones by expressing, “I [do not] want to be trans [and I do not] want to be a girl… I just need to be myself.” Being *myself* in this sense suggests a self-separate from transition and cisnormativity.

### Experiences of Detransition

Several sub-themes were identified within this meta-theme, including *More Like Myself; Alien/ation; Mental, Social and Emotional Well-being; and Corporeal Changes and Impacts*.

#### More Like Myself

Contributors sharing about detransition expressed feeling like a “fraud” or “inauthentic” (Emerson) and even deceitful in the transitioned gender. Furthermore, some contributors were concerned about others’ reactions to their detransition, such as Emerson who suggested they had a lot of anxiety about what was going to happen once they started ‘feminizing.’ They expressed they were fearful of people feeling outraged or as though they had been tricked, and of being met with hostility for having supposedly “deceived people.” Additionally, Emerson suggested they thought transitioning would make them feel authentic in themselves, but rather they felt the opposite, “like a fraud.” Emerson used the term “inauthenticity” in relation to the fear of anger/violence for being perceived to have been lying about their gender. For others, statements such as “being found out” (Delta) appear to invoke a sense of shame about lying, as though the real gender is being purposefully concealed in transition. As Delta explained they were happy for, “not lying to myself or others that… [I am] a female when… [I am] not.” Furthermore, Delta suggested they felt “better and happy,” perhaps even reconciled now they had detransitioned. Lennon post-detransition simply stated “I am feeling more like myself again,” highlighting a sense of not feeling like “myself” and even inauthentic while transitioned. For others, the distance between the transitioned self and the (my)self was experienced as a gap, being not quite present within the world. As Sailor expressed, they had no problems passing as a man, but then felt like they had “put a facade up” between themselves and the world, whereas Rory who had transitioned to “female” stated, “when I was trans… if I slipped up… said the wrong thing, I cease[d] to be female.” Rory’s comment illustrates the punishing and dismissive effects of failing to close the gap between the self and the appearance of a cisnormative gender while transitioned.

#### Alien/ation

Others’ experience of detransition included a sense of feeling *alienated* and like an *alien* in relation to others’ post-detransition. In particular, a number of individuals referred to the effect of corporeal changes made during transition on their experience of belonging. Here Dawson shared their experience considering having their breasts removed: “I feel like… an alien… and I cannot ever go home.” Mason spoke instead more broadly about the experience of being recognized as a woman in a way that aligns with others’ cultural expectations, that it did not feel right to say “I am a woman” as it did not mean the same thing to Mason as it did to others because they were “speaking different languages.” More specifically, Mason suggested that when articulating to others that they are a woman, “they hear I am cisgendered.” For Mason, the absence of transition and detransition in their telling/presentation of their gender is important for their sense of belonging and difference regarding being understood as cisgendered. Conversely, being recognized as trans post-detransition was also an issue as Lennon explained, “[I am] worried people will see me as a trans woman… I… want to have a rest from all the trans stuff… While I am nonbinary… I… want people to see me as a woman… as a person… and not [as] transgender.” While not explicitly talking about feeling like an alien or alienated, Lennon referred to feeling worried and tired of being perceived to be different to cisgender, a form of alien, for example a trans woman or nonbinary.

#### Mental, Social and Emotional Well-being

Supported access to gender-affirming processes, medical procedures, and hormone replacement therapies are a key factor for increasing mental and emotional well-being of trans individuals (James et al., 2016; Schulz, 2018; Swan et al., 2023). While not negating this reality, a number of contributors post-detransition shared about a sense of isolation, exhaustion, anxiety, and even despair regarding the corporeal changes made during transition. Charlie, who had detransitioned 6 months prior, expressed they felt both “alone and tired,” and did not know what to do. Charlie shared that they were somehow “going to have to live in this body” while stressing that “I have no motivation, goals, or hopes… I [do not] know what to do” and felt “stuck” as a consequence. Similarly, George revealed they felt “fucking depressed” and wished they had “never transitioned” and knew the only thing to do now was to accept what they had done.

Additionally, it appears that post-detransition, some contributors continued to experience a desire for and thoughts of being a gender other than that presumed for them at birth. At times contributors reflected they experienced feelings associated with “going mad” (Alexis), while others such as George reported questioning post-transition whether they were “ever going to be a whole man” commenting that they did not “want to keep pretending,” and further questioning post-detransition “how do I live with the idea that if I want to be with a woman, I have to be a man?” The experiences of detransitioning for some appeared to lead to impossible conditions for a flourishing life as feeling concerned and uneasy about their gender continued, despite having detransitioned.

Social well-being was most often reflected through the discussion of relationships, including experiences with dating, coming out, and impacts on friendships. Regarding dating or intimate relationships, of the contributors who had gender-affirmative surgeries, some were concerned that their physical differences, namely genital and breast alteration, may impact the possibility of forming intimate relationships. Phoenix commented, “as a… man who… [had] bottom surgery, am I going to be… single forever? I [cannot]… imagine a woman… wanting to be with me.” Phoenix not only feared they may be unable to form intimate relationships but also seemed to regard themselves as undesirable due to having altered genitals, or the lack of a penis.

For most contributors writing about friendship, it appeared difficult to disclose or come out after detransitioning, some choosing not to disclose for fear of losing friends. Emerson related, “I have made some friends who believe I am cisgender…, and I am afraid to tell them and feel like I will… have to disappear completely.” Ari, who had detransitioned to a cisgender woman similarly stated “coming out… as cisgender was worse. It was humiliating. I… let my friends call me a… man simply because I do not want to tell them.” Others expressed that the reality had been peppered with loss of friendship and isolation; with social media proving an opportunity to stay connected.

#### Corporeal Changes and Impacts

Information regarding corporeal changes, including what to expect when detransitioning, was either very hard to find or nonexistent outside the posts. Contributors shared their experiences of detransitioning in relation to corporeal changes after ceasing hormones and/or post-gender-affirmative surgeries, some specifically asking for others’ lived experiences. Dawson specifically asked “[Do] you get used to not having breasts?” and “has anyone else experienced irregular periods after stopping [hormones]?” while Lennon queried, “will my voice become more feminized?” Contributors also shared photos taken over a time period to describe and tell a story about corporeal changes. For example, Aspen shared photos immediately after ceasing hormones and then after a period of months explaining, “I hated my face… before, but now I feel… better in my own skin.”

A large proportion of detransitioners specifically shared post-detransition changes associated with their hair, mostly regarding its perceived relationship with gender. Miki expressed, “[I] have… detransitioned and… do not know what to do about my long hair. [It is] really long… and I do not want to cut it but it is [a feminine] style.” Dani also asked for “tips on feeling beautiful” post-detransition, and revealed that they were growing out their hair but were not sure what to do until it grew long because they did not feel “feminine with short hair.” For Miki and Dani, hair length and style were associated with femininity and subsequently with beauty. For Miki, long hair signified appearing female but yet reported wanting to be perceived as male but not wanting to cut their hair. For Dani, short hair signified appearing male and this posed a problem as they reported feeling less feminine with short hair. Collectively, these stories about corporeal changes and appearance are important parts of the detransition narrative with real world implications for a sense of gendered embodiment and belonging.

### Prominent Discourses for Detransition

There were two sub-themes related to prominent discourses for detransitioning, namely *Trans Ideology/Trans-Affirming Narrative*, and *Underlying Mental Health Conditions*.

#### Trans Ideology/Trans-Affirming Narrative

Some detransitioners, allies, partners of, and parent contributors used the term trans ideology to describe an experience of being misled and, in a way, convinced that transition was needed. Associated with children and adolescents, contributors frequently commented on being swayed into transition after reading online content, where Zane expressed how children “encounter trans content online and [are] convinced… they have dysphoria” and Reese expressed, “I was influenced online to believe that this, combined with… [being] tomboyish… must mean [I am]… actually a male in a woman’s body.”

Some spoke more generally about being subjected to trans ideology and deceived into transitioning. Blake expressed how the power of simplified messaging about trans ideology coerces teenagers into believing that transitioning is able to completely change them into someone else. Others communicated explicitly about feeling pushed by physicians and therapists into choosing gender-affirming surgeries and/or therapies, raising two concerns. First, the (perceived) quick diagnosis of gender dysphoria and so-called transgenderism, suggesting that physicians/therapists inadequately investigate the causes for gender related distress. This is exemplified by Kyle who shared that on their first meeting with the therapist, they were ready to start Kyle on hormone replacement therapy. Zane similarly recommended, “find a… therapist who will take the time to dig deeper” to a time before feeling “dysphoric and before you wanted to harm yourself with [transition].” The second concern revolves around the belief that physicians advocate for medical-based interventions rather than psychotherapeutic-based interventions. For example, Morgan commented in relation to being diagnosed as gender dysphoric, that they did not know “of any other psychiatric condition that exists that only has one treatment” whereas George remarked they were told by a physician, “you will never be able to feel comfortable… and [need to] drastically alter your body.” While these narratives are concerning regarding diagnosis and supported treatments, notably many others on the site (both detransitioners and those pursuing transition) described experiences of lengthy and thorough processes being required prior to being offered medical-based interventions.

#### Underlying Mental Health Conditions

A common, but less frequent idea within the detransition narratives included the belief that those feeling distressed with the gender presumed for them at birth have underlying mental health conditions and/or hate themselves and are self-harming by transitioning. Here Zane stated, “dysphoria is not a physical disease…, it is a mental state… A synonym for self-hatred… It becomes a mental obsession. Medicalization…, hormones and amputations… will not fix the mind.” In Zane’s statement, it is clear that they believe it is the mind (not body) that needed fixing. Similarly, River indicated that some underlying mental health condition was the cause for feeling distressed when they suggested that trans people think transition will solve their mental health condition, but later realize that being born in the “wrong body” is not the cause of their mental health issues.

While Zane and River were not specific about the mental health issues that might cause individuals to feel distressed about their gender, a frequently mentioned possibility is unresolved childhood trauma. Dawson, sharing about their experience suggested “addressing… childhood trauma… and rejecting gender identity has been much more… healing than anything related to transition ever could have been.” For Dawson, the issue of their distress was not located in concepts of gender identity but rather in the impacts of past trauma.

## Discussion

While the analysis of narratives in social media posts highlights some experiences of detransition similar to those found in other studies, a key difference in findings relates to the methodological approach. Broadly aligning with Butler and Hutchinson (2020), Hildebrand-Chupp (2020), and Littman’s (2021) general call for research involving participant narratives of detransition, this study used a constructionist methodology to explore the underlying social contexts and forces influencing experiences of gender in detransition. As such, gender is not essentialized, and detransition is perceived as a mechanism facilitating one of many possible ways of inhabiting gender. Distinct from other studies, this research has examined and called into question the role of social mores and related cisnormative discourse in shaping gendered embodiment and belonging through detransition.

An array of narratives was identified by those contemplating detransition or who had detransitioned. Many contributors expressed not feeling “myself” or “trans” or “authentic” in the transitioned gender, some suggesting they felt distant from the self. Authenticity seemed to underpin many of these narratives and manifested as an incongruence where the transitioned self was perceived not to be the *true* or *real* self. In addition, authenticity was often imbued with moral value (e.g., where a person’s transitioned gender was evaluated as being inauthentic,) they in turn were deemed fake and even morally corrupt (Gino et al., 2015). Evident within these post-transition narratives of incongruence and inauthenticity are experiences of significant distress that are arguably the result of an unlivable life (Butler, 2004). Relevant to the idea of authenticity, the term “passing” was used to represent being a recognizable gender (in a Butlerian way), resulting in a reprieve and dip into livability. However, for some who transitioned, “passing” rendered the true gender (un)real, invisible, and other; resulting in unlivable lives. Detransitioning in this way appeared to function as a tool of revelation, uncovering as it were the real gendered self. Through the lens of livable lives, the real gendered self received recognition by the self, and/or others that in turn produced a sense of congruence and stability or, as Lennon stated, being “more like myself.”

When questioning how detransition challenged and/or affirmed normative gender, contributors expressed not feeling at home in the transitioned gender as a primary reason for detransitioning. Detransitioning for some resulted in an experience of feeling “better in my own skin” (Aspen); signifying somewhat of a home coming. Conceivably, for some reaching home was more than possible, it was comforting. However, extending Crawford’s (2010) theory of trans architecture, we contend that this home manufactured through detransition may be a site outside of cisnormativity purpose built for its inhabitants. For others, even post-detransitioners, this sense of not being recognized and at home continues to exist. For Mason, the term “woman” engaged post-detransition, did not encapsulate their gender transformation, history, nor culture; failing to speak to all their gender had entailed. Mason alluded to the temporality of home in gender, the hetero/homo-normative space embodied by cisgender. Dawson described feeling “like an alien” post-detransition, an experience of being unearthed or unrooted in gender, but went further to say that home is forever unattainable. This suggests that for some, both transitioning and detransitioning did not automatically signal a return to home. In terms of a trans architecture (Crawford, 2010) this place (of not home) for some, may be explained as the result of a transition from (unattainable) home to unattainable home (and in the case of detransition) to unattainable hetero/homo-cisnormative home.

Talking further of home, Ori asked, “am I recognizable as a woman to other women in lesbian culture when I do not have breasts and have also intentionally removed them.” Their experience speaks of the recognizability of the body within cisnormative discourse post-alteration. What is evident here, is that some who detransition experience real, sometimes severe distress about changes to their corporeal bodies and personal and social affects after gender-affirmative therapies. For them, the consequences of transition and incongruence have been devasting, at times resulting in suicidality.

Narratives reflecting being “pushed” to transition by physicians/therapists/family/ social media espousing trans ideology and/or trans-affirmative models of care are complicated in this context. These narratives appear to reflect that trans-affirmative discourse is the cause of devasting consequences of gender-affirmation leading to detransition. This belief raises questions regarding self-agency and responsibility in relation to informed decision-making especially given the power imbalance between physicians/therapists and their patients. Additionally, this belief is limited in that it fails to question why some are more susceptible to influence than others and essentializes the gender presumed at birth. The gap between providing adequate and even best practice care for those who feel other to the gender presumed for them at birth, while recognizing the monumental impact of transitioning for some who detransition is a vast territory needing prompt and extensive research. In an attempt to bridge the gap, a trans architectural view (Crawford, 2010) considers providing access to gender-affirming therapies that include supporting other ways of constructing gendered homes and contributes to making gendered lives livable.

Most contributors expressed their own stories of detransitioning, often asking questions or seeking support from others with lived experiences. Some were concerned with expected corporeal changes, how to cope and not transition while feeling as though they are not the gender presumed for them at birth, and challenges with relationships and isolation. Others expressed concerns about others’ desire to transition, for the contemplator’s health and well-being, as well as dismay about their choices. Overall, these contributors described the importance of support in understanding why people detransition. Some contributors used the site to express strong views about the role of trans ideology, including social and medical/therapeutic practices in shaping trans bodies. The global social media site thus appeared to provide a platform for expressing frustration, fear, and anger about a sense of powerlessness over gendered outcomes, and shared information and resources to those seeking it. These contributions added to much needed community knowledge and information networks. Notably, as also highlighted in Vandenbussche’s (2021) study, the role of social media for those who detransition is significant in terms of accessing information, establishing connections, belonging and having a safe place to express stories of detransition.

### Conclusion

In conclusion, this paper has shed light on experiences of and narratives emerging from and about detransition, and how these were shared on a global social media site. By navigating stories of detransition, the ways and means by which cisnormative discourses govern, shape, and affect gendered lives were exposed. Further, opportunities for stretching our understanding of detransition and incorporating ways of recognizing gender were offered. Apparent from the findings was that for some, transitioning from the gender presumed at birth may not be the final destination and may represent an uncomfortable abode. By extending Butler’s (2004) concept of livable lives and Crawford’s (2010) notion of trans architecture to encompass detransition, this study has provided a method to envisage detransition—a way of imagining the complexity of normative temporal gendered lives and, advanced the possibility of non-normative understandings of gendered homes, past and present. Reflecting on our work as trans rights and health scholars, allies and clinicians, with trans and detrans lived experiences, this paper has provided a platform for further research in support of working with and for trans, and those who detransition.
